# Acceptance and feasibility of a low-threshold and substitution services-based periodical monitoring system for blood-borne and sexually transmitted infections among people who inject drugs in Germany: a mixed-methods analysis

**DOI:** 10.1186/s12954-024-00977-0

**Published:** 2024-03-14

**Authors:** Amrei Krings, Gyde Steffen, Ruth Zimmermann, the DRUCK 2.0-study group

**Affiliations:** Department of Infectious Disease Epidemiology, Robert Koch Institute, Nordufer 20, 13353 Berlin, Germany

**Keywords:** People who inject drugs, Monitoring, Infectious diseases, Germany, Acceptance, Feasibility

## Abstract

**Background:**

To reach the global elimination goals of viral hepatitis B and C (HBC, HCV), human immunodeficiency virus (HIV) and other sexually transmitted infections as a public health threat by 2030, monitoring is needed. Staff members of drug services and opioid substitution treatment (OST) practices in Berlin and Bavaria recruited clients for a pilot study addressing the respective infections among people who injected drugs (PWID) in Germany, 2021/2022. Participants filled a questionnaire and were tested for HBV, HCV, HIV and syphilis using dried blood spots (DBS). We evaluated the study design to implement a feasible and accepted nationwide periodical monitoring among PWID and serve as an example for the implementation of similar monitoring systems in other countries.

**Methods:**

A mixed-methods design was used, including focus group discussions with study participants and staff members and a semi-quantitative questionnaire filled by the latter. Aspects covered were the setting for recruitment, study preparation for staff members, willingness of clients to participate, the study questionnaire, blood collection and return of results.

**Results:**

The majority (96%) of 668 study participants were recruited in low-threshold services, drug consumption rooms and OST-practices. Flexibility of recruiting study participants during routine work or testing weeks/days was important to the facilities. Collaborations with local AIDS services helped cope with the work load of data collection. The need to train staff for DBS collection was highlighted. Study participants welcomed the testing opportunity in familiar places. Study participants frequently needed assistance to complete the study questionnaire. Return of results was considered as ethically mandatory by staff members but referral to treatment remained challenging.

**Conclusions:**

For a successful monitoring time flexibility and adequate training are essential. Individual benefits for study participants by receiving their test results should be ensured and referral networks with infectiology practices may increase number of infected PWID receiving treatment. Overall, the evaluation confirmed that a monitoring through drug services and OST-practices is feasible and well accepted in Germany. Beyond that it shows important lessons learnt for the implementation in other countries.

**Supplementary Information:**

The online version contains supplementary material available at 10.1186/s12954-024-00977-0.

## Background

In order to reach the goal to eliminate hepatitis B (HBV), hepatitis C (HCV), the human immunodeficiency virus (HIV) and other sexually transmitted infections as a public health threat by 2030, such as defined in the *Global Health Sector Strategy* on Viral Hepatitis by World Health Organization (WHO) [1], the public health response with improved prevention, testing and treatment options needs to be adapted to groups particularly vulnerable for these infections [2]. Especially in countries with low prevalence in the general population, such as Germany, injecting drug use accounts for a large proportion of transmissions [3–5]. Hence, Germany took up the global elimination goals and published a country-specific strategy that prioritizes population groups at high risk for infection for public health measures [6].

An international modelling study estimates that 131,500 (14,000–249,500) people in Germany inject drugs [7]. Portraying the German context a little further, prevention and socio-medical care for people who inject drugs (PWID) are under the responsibility of the federal states and municipalities and very heterogenous across the country. Common services are low-threshold drug services, housing projects for PWID, drug counselling centres and medical practices for opioid substitution therapy (OST) but availability greatly varies. Drug consumption rooms for example exist in only half of the 16 federal states [8]. The number of people using services for PWID is not assessed on a national level. Frequent offers by services for infection prevention include needle exchange, infection counselling and testing opportunities. Testing for PWID in drug services is based on voluntary point of care testing (PoCT) for HIV- and HCV- antibodies, which is legally allowed without the supervision and counselling of a medical doctor. A survey from 2020 among drug services in Germany found that one third of facilities offered PoCT [9].

A first national cross-sectional study on HBV, HCV and HIV among PWID was conducted in 2011–2014 [10, 11]. The results showed high prevalence of HCV and HIV, insufficient HBV vaccination and lacking access to infection testing and care. These finding resulted in several recommendations to expand drug paraphernalia distribution, continue to motivate smoking instead of injection as well as information campaigns on viral hepatitis both for service staff as well as for PWID [12, 13]. Furthermore, a multicentre project to increase testing and linkage to care in low threshold services was conducted [14]. The assessment of the infection prevalence and related behaviour however has not been updated since then.

For surveillance of these parameters and subsequent adaptation of public health measures, in order to reduce the burden of disease and reach the 2030 elimination goals, a continuous and nationwide monitoring of blood-borne and sexually transmitted infections among PWID is needed. This paper reports the results of the pilot study for setting up such a monitoring system in Germany with focus on results regarding feasibility and acceptance.

### DRUCK 2.0

The pilot study DRUCK 2.0 was conducted in Berlin and Bavaria, representing an urban and a rural federal state in Germany. Between 01/06/2021 and 30/04/2022 clients of different kinds of low-threshold drug services and patients in OST practices (from hereon called facilities) were recruited for study participation by the staff of these facilities during their routine work.

Clients or patients were eligible for inclusion if they were 16 + years of age and had injected drugs during the last 12 months. Potential participants were informed about the study and had to provide written informed consent. Participation included a paper-based questionnaire with 20 main questions and 19 conditional questions on sociodemographic characteristics, risk and protective behaviour and access to testing and care for HBV, HCV, HIV and syphilis that was written in simple language and available in 12 different languages. Furthermore, a blood sample was collected for HBV, HCV, HIV and syphilis testing in a central laboratory. This was either capillary blood for dried blood spots (DBS) in low-threshold services or venous blood in OST practices. Study participants received a 10 Euro incentive voucher.

Recruiting facilities were identified through previous partnerships, networks of collaboration partners, online search and a list from the Association of Statutory Health Insurance Physicians for OST practices, and invited for participation 3 months prior to the study start. Staff members of participating facilities were trained for pre- and post-test counselling, if necessary, and data-/blood collection though virtual and (optional) on-site training courses. A checklist filled out for each participant with all relevant steps of the study and standard operating procedures were available. The facilities had the flexibility to choose whether recruitment was done as testing days/weeks or continuously over a three months period of time. A coordinating study team at the Robert Koch Institute provided the facilities with the study materials and was available to support problem solving.

In order to compare different study modalities, the facilities were assigned to one of the following three study arms, depending on their availability of testing services and medical doctors: the laboratory test results (LTR) were not returned to the study participants, but participants were referred to routine testing (study arm 1), the LTR were not returned to the study participants, but participants were offered antibody based PoCT for HCV and HIV (study arm 2), the LTR were returned to the participant through the facilities (study arm 3). Additionally, participants could spontaneously decide whether they wanted assistance by staff members to fill out the study questionnaire. Language mediation via telephone was available.

The aim of DRUCK 2.0 was to find the most accepted and feasible study design for a nationwide and periodical monitoring of blood-borne and sexually transmitted infections among PWID that can be implemented in Germany but also serve as an example for the implementation in other countries. We therefore conducted an evaluation after the data collection focussed on the central questions of where participants for a periodical monitoring can be recruited, how this should be done and who can be reached when using the anticipated study design.

## Methods

This analysis shows the results of a mixed-methods approach to evaluate the feasibility and acceptance of the applied DRUCK 2.0 study design. In detail, the evaluated aspects were: (i) recruitment through drug services and OST practices during their routine work, (ii) pre-study training for staff members as well as practical and organizational aspects, (iii) questionnaire and potential need for assistance, (iv) translations and language mediation, (v) blood collection in the various recruiting services, and (vi) the different options to return the central laboratory test results.

Each facility filled out a semi-quantitative exit questionnaire on the aspects mentioned above (Additional file 1: Exit questionnaire). Information on the recruiting facilities' name and type, performance of HCV and HIV PoCT (only study arm 2), the need for translation and assistance for filling out the questionnaire was documented for each participant. Additionally, facilities were asked to document the sex, estimated age and perceived German language skills of clients who declined participation (non-responder analysis). The central laboratory documented the type of blood sample and, if applicable, the quantity and quality of DBS for each participant.

Quantitative descriptive analysis of the exit questionnaire and other collected information were conducted using STATA 17. The variables analysed from the exit questionnaire included expenditure of time needed, feeling of being prepared for the study, rating of on-site visits prior to study start, length and complexity of study questionnaire, type of assistance provided for study questionnaire, feasibility of DBS, acceptance of PoCT instead of laboratory result return, estimated proportion of laboratory results picked up by study participants. Moreover, type of recruiting facility, participants’ need for assistance filling out the questionnaire, language of questionnaire, type of blood sample (DBS, venous), number and quality of DBS, use of a PoCT for HCV/HIV were analysed. Frequencies and proportions were calculated.

**Fig. 1:**
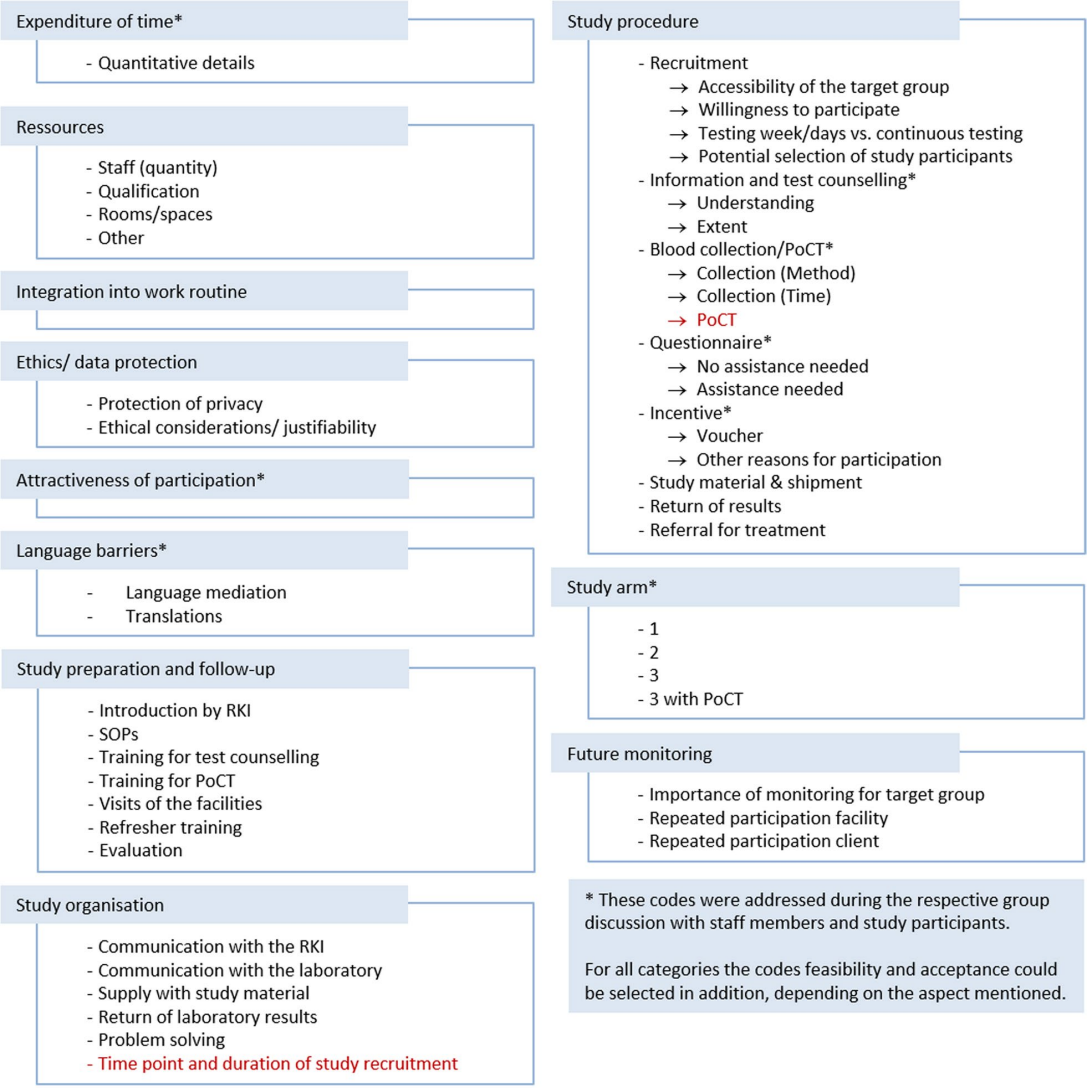
Categories with deductive (black)/ inductive (red) content analysis codes of the group discussions with facilities/study participants. *PoCT* Point of care test, *RKI* Robert Koch Institute, *SOPs* Standard operating procedures

Additionally, three focus group discussions were conducted in Berlin and Bavaria with staff members of facilities and four with study participants using a semi-structured interview guide covering the above-mentioned aspects (Additional file 2: Interview guide). Group discussions were moderated by the DRUCK 2.0 study team. Staff members provided their oral consent to audio-record the focus group discussion and received no financial incentive to participate in the group discussion. Study participants provided their written consent for audio-recording and received a 10€ incentive voucher. The recordings were transcribed verbatim. Analysis was based on the qualitative content analysis guidelines by Philipp Mayring in a deductive-inductive manner using MAXQDA© 2022 (VERBI Software GmbH, Berlin, Germany) [15]. Deductive categories resulted from the semi-structured interview guides and inductive categories from the answers given by staff members or study participants (Fig. 1).

Ethical approval for the DRUCK 2.0 study, including the evaluation, was provided by the Medical Chamber Berlin (ETH-51/10).

## Results

In total, 25 facilities (20 drug services and 5 OST practices) in 7 cities agreed to participate in the DRUCK 2.0 study and recruited 668 clients from June-October 2021 in Berlin and November 2021–April 2022 in Bavaria.

Overall, 24 facilities filled the exit questionnaire. 25 staff members of 18 facilities and 23 study participants joined the focus group discussions. A checklist was received for 632, a questionnaire for 633 and a blood samples for 666 study participants.

### Setting and workload

Seventy percent (468/668) of the study participants were recruited in low-threshold drug services, 17% (113/668) in consumption rooms, nine percent (63/668) in OST practices and four percent (24/668) in drug counselling centres and housing projects. Two additional OST practices agreed to participate in the study but did not recruit any clients. Overall, it was difficult to recruit OST practices for the study. Many were involved in the COVID-19 vaccination efforts or reported not having clients who injected drugs during the last 12 months. Drug counselling centres and housing projects also reported a reluctance of clients to confirm current injecting drug use and therefore recruited only few participants. As reasons for the facilities to participate in the study, staff members mentioned that they wanted to enable their clients to have access to laboratory testing. Another reason was the possibility to support data collection that would help adapting local offers according to their clients’ needs. Staff of several facilities reported having specifically participated to support the implementation of a periodical monitoring among PWID or wanted to benefit from the training opportunities for PoCT and test counselling in order to implement routine test offers in their facility in future. As a positive side effect, some facilities reported gaining new clients and intensifying their contact with the clients by conducting the study.

*“Through the study, we gained a few new clients from outside for the facility, which [I] found cool.”* (Quote by a staff member during the facility group discussions; Translated from German by study team)

Overall, the time needed to conduct the study was rated as “not high” by 46% (11/24), “somewhat too high” by 33% (8/24) and “too high” by 21% (5/24) of the facilities. During the facility group discussions, staff members reported having underestimated the effort needed, but also that unforeseen problems, such as shortfall of personnel and increased workload due to COVID-19 prevention measures, impeded the study implementation. Integration of the study in the routine work was mentioned to have been more complicated than expected, and many staff members reported that this is why the flexible choice between recruiting clients continuously or during testing days or weeks according to the facilities’ capacities was very important. A time period for recruitment between 3–4 months was mentioned as a feasible time frame by staff members during the facility group discussions. Some facilities independently collaborated with local AIDS services that came to the facilities and conducted the blood collection and (if needed) PoCT, while the facilities’ staff members recruited their clients and assisted with the questionnaire. This was mentioned to have worked well and reduced the work load.

In the facility group discussions, staff members expressed that they appreciated having been able to contact the study team in case of questions. Moreover, the prepacking of study materials, which was only conducted in Bavaria, was helpful to fasten the organisational steps in the facilities.

### Study preparation

Of all the facilities with respective responses, 43% (10/23) rated their feeling of being prepared for the study as “very well”, 48% (11/23) as “well” and 9% (2/23) as “not so well”. Especially the on-site visits prior to the study start with hands-on training of blood collection were rated as “very helpful” by 61% (11/18) or “helpful” by 39% (7/19) of the responding facilities. During the facility group discussions, the virtual introduction to the study and the checklist with an overview of every step that needed to be done per study participant were complimented. However, the facilities mentioned that they had time and personnel constrains due to the study and wished to receive earlier notice prior to the next study start in order to adjust their schedule accordingly. A short information with an overview of how many meetings the study participation (including training, on-site visit by RKI, implementation of the study and evaluation) entailed would have been helpful in order to anticipate the effort needed was suggested.

### Acceptance of participation by clients

During the facility group discussions, staff members from low-threshold services and consumption rooms reported a good access to clients fulfilling the study’s inclusion criteria and a high willingness to participate.

*“[…] recruitment was easy for us and could have simply continued, because we are here […] in the middle of the scene.”* (Quote by a staff member during the facility group discussions; Translated from German by study team)

Counselling centres and housing projects reported more difficulties with recruitment, because their clients either had not injected during the past 12 months, were not admitting that they had injected or came for fixed appointments and did not have extra time to participate. Similar feedback was received from OST practices, who reported that they could have recruited more patients if people who ever injected were included.

As only very few facilities returned the counting of clients who declined study participation, most of them with the note that staff members were unable to fill them out reliably, we were not able to compare clients who were willing to participate with those who declined participation.

During the group discussions with study participants they reported having a good trust in the facilities and their staff members. Many would not have participated if recruitment had taken place in a clinic. As reasons for participation many mentioned the incentive, but also the opportunity to check their infection status and receive counselling.

*"Yes, well, so for me it was important to simply check what the situation is.”* (Quote by a study participant during the participants group discussions; Translated from German by study team)

The incentive (10€ supermarket voucher) was generally well received but cash would have been preferred. Some reported feeling patronized by receiving a voucher instead of cash.

### Study questionnaire

The study questionnaire was rated with regards to the length as “a bit too long, but feasible” by 71% (17/24) of the facilities and with regards to comprehensibility as “a bit complicated, but still understandable” by 63% (15/24) of the facilities in the exit questionnaire. The time it took to complete the questionnaire was not systematically collected, however staff members reported during the facility group discussions that their clients needed around 10–20 min and that length of the questionnaire was acceptable. The content of the questionnaire was complimented by staff members and was many times used as the introduction to a general counselling with the clients. Some even reported that it enabled them to intensify their relation to the clients.

*“I found it super exciting, that through the questionnaire we were able to build a different contact with our clients. Because it was for the study questionnaire, the people all of a sudden started to talk. Through this, I could establish a closer contact to many study participants.”* (Quote by a staff member during the facility group discussions; Translated from German by study team)

However, many staff members pointed out that some questions, especially conditional questions asking for example for the consumption or usage of shared needles and syringes during a specific time period, were too complex. Overall, 43% of the study questionnaires were answered without assistance by staff members and 57% needed assistance. In the exit questionnaire 70% (14/20) of the facilities reported giving assistance by reading out the questions and answers and 30% (6/20) further explained the questions and answers.

The questionnaire was mainly used in German (89%; 593/668); other translations used were Russian (5.2%; 16/668) English (2.4%; 10/668) Polish (1.2%; 8/668) Arabic (0.8%; 5/668) and Farsi (0.2%; 1/668). The facilities did not report any additional translations that may have been needed throughout the study. Although not frequently used, the availability of translated study documents was seen as very important during the facility group discussions. In contrast, the language mediation via telephone was not used by any facility. Staff members described that they preferred using their own language mediators that are part of the facilities’ staff. It was commented that having an external person not physically present but via telephone in sometimes very noisy environments was not feasible.

*“Via telephone […], this does not work because of the noise. Language mediation in person is better, especially for intimate questions. We also felt more comfortable with a person as a language mediator.”* (Quote by a staff member during the facility group discussions; Translated from German by study team)

Moreover, language mediation would have already been needed to address the clients in order to invite them for participation and that approaching clients with a third person on the telephone would not have been perceived well.

### Blood collection

Overall, 94% (625/668) of the samples were DBS and 6.4% (43/668) venous blood samples. The median number of blood spots collected were 8 (IQR: 8–12 spots). Overall, 51% (314/618) of the spots were of good, 34% (213/618) of moderate, 14% (88/618) of poor and 0.5% (3/618) of insufficient quality. In Berlin blood collection via DBS was still problematic. During the recruitment period in Berlin facilities called and reported having problems with the lancets. It was also mentioned during the facility group discussion in Berlin that the number of spots needed was too high and it took a lot of time to collect sufficient amounts of blood. For the recruitment in Bavaria the lancets were replaced and more time was reserved for hands-on training of the blood collection during the study preparation. Furthermore, the number of spots needed was reduced for the recruitment period in Bavaria. In the exit questionnaire the feasibility of DBS was rated as “good” by 36% (4/11), "not very good” by 55% (6/11) and “poor” by 9% (1/11) of the facilities in Berlin. None of the facilities in Berlin rated the DBS feasibility as “very good”. In Bavaria it was rated as “very good” by 22% (2/9) and as “good” by 78% (7/9) of the responding facilities. None of the facilities in Bavaria rated the feasibility of DBS as “not very good” or “poor”. Besides from the training some staff members noted during the facility group discussions that the blood collection required quite close body contact, which was normally not part of their work and was perceived as uncomfortable.

*“For some people it is for sure already too intimate to stroke a drop of blood from somebodies’ hand. Well, you need to, it is quite close […]”* (Quote by a staff member during the facility group discussions; Translated from German by study team)

Some suggested doing the finger prick, but letting study participants press their fingers to drop the blood on the filter cards. During the participant group discussion, it was reported that blood collection was less painful than expected. Few participants even suggested that they could have also collected venous blood themselves. This however was not agreed upon by the others.

### Return of results and PoCT

Regarding return of the test results, two facilities (9%) chose study arm 1, 14 (61%) study arm 2, and ten (43%) study arm 3. Three facilities (13%) switched between the study arms, conducting study arm 2 in collaboration with a local AIDS help service as part of a testing day and otherwise study arm 1 or 3.

Of all participants recruited in study arm 2 (n = 247), 45% (111/247) did not make use of an HCV- and 18% (44/247) of an HIV- PoCT. However, the participants’ acceptance of the PoCT in study arm 2 was still rated as “very good” by 15% (2/13), “good” by 54% (7/13) and “rather poor” by 31% (4/13) of the facilities in the exit questionnaire. The facilities were also asked how well the participants accepted a PoCT compared to a return of the laboratory results. Here, half (5/10) of the respective facilities responded that this was criticized by some clients, whereas the other half (5/10) responded that there was no or hardly any critique. During the facility group discussions, it was highlighted that the PoCT offer was nevertheless important, especially in low-threshold settings without medical doctors for return of the results.

*“I find it good, because drug users perhaps also do not have the possibility, often do not go to the doctor for many years, and then to take the chance, I find it a good opportunity, actually.”* (Quote by a staff member during the facility group discussions; Translated from German by study team)

A disadvantage discussed was the fact that a reactive PoCT results requires laboratory confirmation, and referral for further testing can be difficult.

However, staff members attending the facility group discussion also stated that they find it ethically important to return the laboratory results to the study participants, even though this is difficult for some facilities due to the lack of medical doctors. Some staff members recommended collaborations with medical doctors of local AIDS help services or OST practices or fixed consultation hours during which a medical doctor was available. Nevertheless, difficulties to return laboratory results were reported and high legal barriers in Germany, requiring a medical doctor to return laboratory results, were criticized. Further, the laboratory sent back the test results to facilities only within 10 days, which was assessed to be too long by the facilities.

*“This does not make sense to have low-threshold testing and high-threshold notice.”* (Quote by a staff member during the facility group discussions; Translated from German by study team)

In the exit questionnaires, half of the facilities (3/6) in study arm 3 with respective responses estimated that under 50% of the results were collected by participants.

Following a reactive PoCT or positive laboratory results, the referral to a medical practice was reported to be difficult. It was described that although many study participants were motivated at first, some ended up not starting their treatment or did not have health insurance covering treatment, due to their non-legal resident status in Germany. However, several facilities did report positive experiences and mentioned that others started treatment after their study participation.

## Discussion

The evaluation of the DRUCK 2.0 pilot study provided valuable insights into the acceptance and feasibility of the various study modalities for a future periodical monitoring of infections and related behaviour among PWID.

Recruitment through low-threshold services or needle and syringe programs has also been described in other monitoring systems and countries [16–18]. Although the workload due to the data collection is reported to be high, time flexibility and, in our example, collaborations with local AIDS help services support the highly successful recruitment in these places. From our view, limiting the overall recruitment period to 3–4 months per data collection round is helpful for the facilities not to lose thread. A longer period of notice ahead as well as sufficient training opportunities (especially hands-on training of blood collection) for staff members are essential, as the majority of staff members in low threshold services are social workers, who are usually not used to medical procedures. However, drug consumption rooms in Germany for example have medical staff (e.g. nurses) among their team and recruitment may greatly benefit from their experience for the blood collection [8]. In this pilot study especially, counselling and housing services have less medical personnel and the staff are more reserved towards close body contact with clients. Moreover, in these services as well as in OST-practices recruitment is more challenging due to less clients, who report recent drug injection and results in low numbers of recruitments.

The pilot study was conducted during the second and third year of the COVID-19 pandemic. Especially during the first year of the pandemic facilities reported having been restricted in their routine work through the infection prevention measures and lockdowns [19]. Some of these restrictions still affected the work at the time of the pilot study. In addition,especially OST-practices were deeply involved in the COVID vaccination efforts at the time of the pilot study [20]. Nevertheless, with a light adaption of the study design (venous blood collection, survey during waiting time) OST-practices were able to recruit 9% of the study participants and should not be underestimated as a setting for recruitment. With participants recruited in OST-practices it would be possible to assess the coverage of infectious disease testing and treatment among PWID regularly seen by OST doctors.

Conducting the recruitment in low-threshold drug services was also well perceived by the study participants and in line with the recommendations given by the consolidated guidelines on HIV, viral hepatitis and STI prevention, testing, treatment and care for key populations published by WHO [2]. PWID face a lot of stigma and other barriers that reduces their access to regular health services. Bringing the monitoring into familiar places increases the likelihood for PWID to participate and contributes to low-threshold prevention. However, this includes a return of the test results, which is discussed below.

It remains unclear how representative the study population recruited during DRUCK 2.0 is for PWID in Germany. Recruitment was based on two convenience clusters. At the first level of clustering facilities willing to recruit were included and at the second level clients who visited the respective facilities. One limitation with regards to the selection of facilities was that there is no central registry of drug services in Germany. The registry for OST-practices did not yield in suitable practices for the study, since many have the license but do not conduct OST in reality. This could lead to a selection of very motivated facilities, known because of their engagement for infectious diseases among PWID, and potentially already offering a number of prevention and testing measures to their clients. Moreover, clients who are better integrated in a support network are more likely to participate in the study. The facilities did not return the non-responder list and therefore, a comparison of those not willing to participate to those participating was not possible. This can also be seen as an indicator that facilities can only recruit a convenience sample during the routine work and that random recruitment of clients may cause a work load that is too high.

In the pilot study we only included PWID with recent drug injection. However, during recent years a lot of effort was invested into the promotion of inhalative opiate consumption as opposed to injection in Germany and changing consumption patterns were already observed and reported, that may also be visible in other countries [21, 22]. This will lead to less PWID fulfilling the inclusion criteria in the future and may support the consideration if inclusion of only recent drug injectors maps the current epidemiological situation correctly. Moreover, OST-practices reported having few patients who injected during the last 12 months, but it can be discussed if recent consumption is openly addressed. This is an important factor that should be discussed based on the countries’ situation, when implementing a monitoring system.

The study questionnaire was established based on the behavioural indicators published by the European Monitoring Centre for Drugs and Drug Addiction (EMCDDA), the study questionnaire from the first DRUCK study conducted in 2011–2014 and the questionnaire used by the UK Unlinked Anonymous Monitoring Survey of PWID [23]. Especially conditional questions targeting specific time periods (e.g. last 30 days) were assessed as too complicated and need to be simplified. Simplification needs to be done, even if this results in reporting less indicators. Around half of the questionnaires were filled out with assistance of a staff member, even though all questions were translated into plain language. This may introduce bias of social desirability to the responses. With a simplified questionnaire, the proportion of self-filled questionnaire can be increased, which may reduce the bias. Generally, studies have shown that using audio-computer-assisted self-interviewing helps in reducing social desirability bias [24]. However, in this study paper-based questionnaires were used, which made it more feasible in outreach settings. Offering assistance for participants who are not able to read or understand the questions is still crucial. Although this means a high workload for staff members, a positive side effect is, that assisting the questionnaire can be used as a good opportunity for counselling.

Language mediation was essential to include also non-German speaking PWID. Surprisingly, the offer of translation via telephone was not used by any facility. In contrast, in-person language mediation by staff members was better accepted and more feasible for staff members and participants. This may lead to exclusion of participants whose languages are not spoken at the respective facility. However, the study documents were available in the most frequently spoken languages, and may help to reduce barriers in this regard.

DBS from capillary blood are widely used for blood collection in studies [11, 16] and have shown benefits for low-threshold settings [25]. A major advantage is that capillary blood can also be drawn by non-medical personnel or participants themselves. Still, this method requires training for the staff members in order to improve the quality of DBS collected and reduce hesitation. In medical practices, the use of venous samples is faster and more convenient for the personnel and should therefore be an alternative option to increase participation of OST-practices in a monitoring.

A main point of discussion is the return of the test results in surveys. Other monitoring systems use study designs without return of the laboratory results [16]. In DRUCK 2.0, the study arms that returned the results to participants were preferred for ethical reasons, even though less than half of the laboratory results were picked up by the clients and some facilities struggled to provide medical counselling. In order to solve the latter, many low-threshold services intensified collaboration with OST-practices. This collaboration may have the additional positive effect of decreasing barriers to OST.

While PoCT were highly accepted by clients in study arm 2, some requested their laboratory result in addition. Considering the increasing likelihood of a resolved HCV infection with increasing years of injecting drug use and due to improved therapy options, antibody-based HCV PoCT becomes less informative as it cannot distinguish between active and resolved infections. This may also be reason for the lower uptake of HCV—compared to HIV PoCT. In addition, many low-threshold services already offer HCV/HIV PoCT but PCR PoCT is still scarce due to high costs.

A major obstacle remains the referral of positively tested participants for treatment. Even if treatment of hepatitis C became a lot easier in recent years due to the introduction of direct-acting antiviral drugs (DAA), PWID may need support to find a respective practice for treatment, organize an appointment and realize this appointment [9]. Pilot projects in some of the participating facilities, showed that employing assistants for these tasks helped overcoming these barriers [26]. Although health insurance is mandatory in Germany, some PWID may have no access to health insurance, e.g. due to a lacking legal status or recent release from prison and hence no coverage of treatment costs [9]. In Germany, local initiatives may cover the treatment costs, however often result in high bureaucratic barriers.

## Conclusions

The results of this pilot study and its’ evaluation provide valuable insights and lessons learnt to set up a future monitoring system in Germany and may support this process in other countries as well, in order to monitor the progress towards the global elimination of viral HBC, HCV, HIV and other sexually transmitted infections as a public health threat by 2030.

In our case, the monitoring system will be set up with low-threshold services and OST-practices as recruiting facilities. A central coordination, responsible for the recruiting and training of facilities, preparation of the study materials, analysing and reporting of the results as well as being available for problems occurring will be located at the Robert Koch Institute.

To gain more representative results and engage political stakeholders, the commissioners for drug addiction in all of the 16 federal states in Germany, who engage with facilities on a regular basis, will be invited for a meeting prior to the start of the monitoring and ask to promote participation in their networks.

The monitoring will be conducted as a periodical cross-sectional data collection over a 3–4 months time period every 2–3 years, with notice to the facilities 6 months prior to the recruitment start. Facilities will be able to flexibly choose whether they recruit participants continuously during the recruitment period or organize designated testing days or weeks, possibly with local AIDS help services supporting them.

One of the major changes for the future monitoring system compared to the pilot study will be the inclusion of ever injectors, in addition to recent injectors.

Participants will receive a paper-based questionnaire and the choice to ask for assistance, if needed, will remain part of the study design. The questionnaire will be simplified and shortened, despite the fact that some indicators cannot be answered anymore. Translations in plain language and the most frequently spoken languages, as reported by the facilities, will remain available, however language mediation via telephone will not be offered for the monitoring.

For blood collection the monitoring will allow venous blood collection for OST-practices and capillary blood collection with DBS for low-threshold services. Sufficient training for capillary blood collection needs to be scheduled as part of the study preparation.

Last but not least, the return of the results remains an important aspect. In the future monitoring, all participants will be given the opportunity to receive their full laboratory result. Facilities will be asked to prepare for the study by collaborating with medical doctors, for example from OST-practices or local AIDS help services. Also, networking with infectiology practices needs to be intensified. This will be supported by the central coordination team and is crucial for treatment referral of positively tested participants.

### Supplementary Information

**Additional file 1**: Exit questionnaire.

**Additional file 2**: Interview guide.

## Data Availability

The datasets used and/or analysed during the current study are available from the corresponding author on reasonable request.

## Abbreviations

DAA: Direct-acting antiviral drugs
DBS: Dried blood spots
HBV: Hepatitis B Virus
HCV: Hepatitis C Virus
HIV: Human Immunodeficiency Virus
LTR: Laboratory test result
OST: Opioid substitution treatment
PWID: People who inject drugs
PoCT: Point of care test(ing)

## References

[1] World Health Organization. Global health sector strategy on viral hepatitis 2016–2021. Towards ending viral hepatitis. Geneva: World Health Organization; 2016.

[2] World Health Organization. Consolidated guidelines on HIV, viral hepatitis and STI prevention, diagnosis, treatment and care for key populations. Geneva: World Health Organization; 2022. 36417550

[3] Trickey A, Fraser H, Lim AG, Peacock A, Colledge S, Walker JG (2019). The contribution of injection drug use to hepatitis C virus transmission globally, regionally, and at country level: a modelling study. Lancet Gastroenterol Hepatol.

[4] Steffen G, Behnke A, Dudareva S, Hommes F, Krings A, Kollan C, et al. Virushepatitis C im Jahr 2021. EpiBull. 2022;38/2022:19.

[5] Falla AM, Hofstraat SHI, Duffell E, Hahné SJM, Tavoschi L, Veldhuijzen IK (2018). Hepatitis B/C in the countries of the EU/EEA: a systematic review of the prevalence among at-risk groups. BMC Infect Dis.

[6] Bundesministerium für Gesundheit, Bundesministerium für wirtschaftliche Zusammenarbeit und Entwicklung. Strategie zur Eindämmung von HIV, Hepatitis B und C und anderen sexuell übertragbaren Infektionen. Bis 2030 - Bedarfsorientiert · Integriert · Sektorübergreifend: Bundesministerium für Gesundheit; 2016. Available from: https://www.bundesgesundheitsministerium.de/themen/praevention/gesundheitsgefahren/hiv-hepatitis-und-sti/bis-2030.html, http://www.bundesgesundheitsministerium.de/fileadmin/Dateien/5_Publikationen/Praevention/Broschueren/Strategie_BIS_2030_HIV_HEP_STI.pdf

[7] Hines LA, Trickey A, Leung J, Larney S, Peacock A, Degenhardt L (2020). Associations between national development indicators and the age profile of people who inject drugs: results from a global systematic review and meta-analysis. Lancet Glob Health.

[8] Deutsche AIDS Hilfe e.V., Drogenkonsumräume. Standorte und Informationen zu Konsumräumen in Deutschland: Deutsche AIDS Hilfe e.V.; 2023. Available from: https://www.drogenkonsumraum.de/de

[9] Schulte B, Jacobsen B, Kuban M, Kraus L, Reimer J, Schmidt CS, Schäffer D, Umsetzung von Testung, Diagnostik und Behandlung der Hepatitis C in Einrichtungen der niedrigschwelligen Drogenhilfe in Deutschland–eine Querschnittsbefragung. Suchttherapie. 2022(EFirst).

[10] Robert Koch-Institut. Abschlussbericht der Studie „Drogen und chronischen Infektionskrankheiten in Deutschland“ (DRUCK-Studie) Berlin: Robert Koch Institut; 2016. Available from: http://www.rki.de/DE/Content/InfAZ/H/HIVAIDS/Studien/DRUCK-Studie/Abschlussbericht.pdf

[11] Wenz B, Nielsen S, Gassowski M, Santos-Hövener C, Cai W, Ross RS (2016). High variability of HIV and HCV seroprevalence and risk behaviours among people who inject drugs: results from a cross-sectional study using respondent-driven sampling in eight German cities (2011–14). BMC Public Health.

[12] Deutsche AIDS-Hilfe. Empfehlungen für die Vergabe von Drogenkonsumutensilien Berlin: Deutsche AIDS-Hilfe e.V.; 2018. Available from: https://www.aidshilfe.de/sites/default/files/documents/empfehlung_konsumutensilien_final.pdf

[13] Deutsche AIDS-Hilfe EV, Institut für Suchtforschung. SMOKE-IT! - Unterstützung zur Veränderung der Drogenapplikationsform (von intravenös zu inhalativ) mittels neuartiger Präventionstools sowie medialer und personaler Interventionen. Frankfurt am Main: Fachhochschule Franfurt am Main; 2010.

[14] Gerlich M, Dichtl A, Graf N, Abschlussbericht zum Modellprojekt HIV? Hepatitis? Das CHECK ich! Köln: Bundeszentrale für gesundheitliche Aufklärung, BZgA 2020 Juli 2020.

[15] Mayring P, Qualitative Inhaltsanalyse. Grundlagen und Techniken. 13. überarbeitete Auflage ed. Weinheim und Basel: Beltz; 2015 20.07.2022

[16] Public Health England. Unlinked Anonymous Monitoring (UAM) Survey of HIV and viral hepatitis among PWID: 2022 report. London; 2022 August 2022.

[17] Kagstrom E, Lannergard A, El Khosht J, Lorelius P, Manflod J, Stromdahl S (2023). Prevalence, risk factors, treatment uptake and treatment outcome of hepatitis C virus in people who inject drugs at the needle and syringe program in Uppsala, Sweden. Harm Reduct J.

[18] Iversen J, Wand H, McManus H, Dore GJ, Maher L (2023). Incidence of primary hepatitis C virus infection among people who inject drugs in Australia pre- and post-unrestricted availability of direct acting antiviral therapies. Addiction.

[19] Krings A, Steffen G, Germershausen C, Zimmermann R (2020). Auswirkungen der COVID-19-Krise auf Präventionsangebote zu durch Blut und sexuell übertragenen Infektionen bei Drogengebrauchenden. EpiBull.

[20] Bundesministerium für Gesundheit. National COVID-19 Vaccination Strategy. Strategy for the continued delivery and evaluation of vaccination agains SARS-CoV-2 in Germany (updated): Bundesministerium für Gesundheit; 2021. Available from: https://www.bundesgesundheitsministerium.de/fileadmin/Dateien/3_Downloads/C/Coronavirus/Impfstoff/National_COVID-19_Vaccination_Strategy_June_2021.pdf

[21] Suchtkooperation NRW, Jahresbericht 2021 Drogenkonsumräume in Nordrhein-Westfalen. 2021.

[22] Stöver HFS, Drogenkonsumraum-Dokumentation. Auswertung der Daten der vier Frankfurter Drogenkonsumräume. Jahresbericht 2021. Frankfurt am Main: Institut für Suchtforschung (ISFF), Frankfurt University of Applied Sciences; 2022.

[23] European Monitoring Center for Drugs and Drug Addiction. Drug-related infectious diseases (DRID) toolkit: European Monitoring Center for Drugs and Drug Addiction; 2013. Available from: https://www.emcdda.europa.eu/publications/manuals-and-guidelines/drid-toolkit_en

[24] Des Jarlais DC, Paone D, Milliken J, Turner CF, Miller H, Gribble J (1999). Audio-computer interviewing to measure risk behaviour for HIV among injecting drug users: a quasi-randomised trial. Lancet.

[25] Coats JT, Dillon JF (2015). The effect of introducing point-of-care or dried blood spot analysis on the uptake of hepatitis C virus testing in high-risk populations: a systematic review of the literature. Int J Drug Policy.

[26] Condrobs EV, Sachbericht 2022. Health Advisor Projekt München. 28.02.2023: condrobs e.V.; 2023.

