# Supplementary material for: Acceptance and feasibility of a low-threshold and substitution services-based periodical monitoring system for blood-borne and sexually transmitted infections among people who inject drugs in Germany: a mixed-methods analysis
Source: Harm Reduct J. 2024 Mar 14;21:62. doi: 10.1186/s12954-024-00977-0 (PMC10938743; doi:10.1186/s12954-024-00977-0)
Supplement: Supplementary file 1 — Additional file 1: Exit questionnaire. [file 12954_2024_977_MOESM1_ESM.pdf]

Evaluation Exit Questionnaire (translated from German)

Overall

Only for study arm 2&3

1) Where were the participants mainly recruited in your organisation? (multiple answers possible)

O Low-threshold drug service
O Consumption rooms
O Drug counselling centres
O Housing projects
O Street work
O Other: ____

Preparation for the study

2) Overall, how well did you feel prepared for conducting the study?

O very well
O well
O not so well
O poorly

If you did not feel well prepared, do you have any suggestions for improvement?
____
____
____

3) How would you rate the amount of time and personnel needed preparing for participation in the study (participation in training, introduction, visit to the RKI)?

O not at all high
O not high
O slightly too high
O much too high

Only for study arm 2&3

4) If you have taken part in the training course on test counselling and rapid test implementation, how would you rate it as preparation for the study?

**Scope**	**Information content**	**Benefit for conducting the study**
O too short	O very good	O very good
O adequate	O good	O good
O too long	O rather poor	O rather poor
	O poorly	O poorly

O We did not take part in the training courses

Comment if applicable (free text)

____

____

____

Only for study arm 2&3

5) If you did not take part in the training course on test counselling and rapid test implementation, what was the reason for this? (e.g. already trained, no time, travelling too far, etc.)

____

____

____

6) How do you rate the virtual introduction as preparation for the study??

**Scope**	**Information content**	**Benefit for conducting the study**
O too short	O very good	O very good
O adequate	O good	O good
O too long	O rather poor	O rather poor
	O poor	O poor

O We did not take part in the introduction

Comment if applicable (free text)

____

____

____

7) How do you rate the on-site visits of the RKI study team as preparation for the study?

O very helpful
O helpful
O not very helpful
O not helpful at all

What was particularly/not very helpful? (free text)

____
____
____

Only asked in Bavaria

8) How do you rate the virtual refresher meeting in January as support for the study programme?

**Scope**	**Information content**	**Benefit for conducting the study**
O too short	O very good	O very good
O adequate	O good	O good
O too long	O rather poor	O rather poor
	O poorly	O poorly

O We did not take part in the refresher meeting

Comment if applicable (free text)

____
____
____

9) How do you rate the SOPs as a tool for conducting the study?

**Scope**	**Comprehensibility**	**Usefulness for the study**
O too short	O very good	O very good
O adequate	O good	O good
O too long	O rather poor	O rather poor
	O poorly	O poorly

Comment if applicable (free text)

____
____
____

10) Are there areas of the study for which there was insufficient preparation from your perspective (e.g. study procedure, blood sampling, questioning, test result, etc.)?

O yes
O no

If yes, which ones? (free text)

____
____
____

Organisation and implementation

11) How would you rate the time it took your organisation to conduct the study (recruitment of participants, testing, survey)?

**Total**	**Recruitment**	**Testing**	**Questionnaire**
O not high at all	O not high at all	O not high at all	O not high at all
O not high	O not high	O not high	O not high
O somewhat too high	O somewhat too high	O somewhat too high	O somewhat too high
O too high	O too high	O too high	O too high

12) How would you rate the personnel effort it took your organisation to conduct the study (recruitment of participants, testing, survey)?

**Total**	**Recruitment**	**Testing**	**Questionnaire**
O not high at all	O not high at all	O not high at all	O not high at all
O not high	O not high	O not high	O not high
O somewhat too high	O somewhat too high	O somewhat too high	O somewhat too high
O too high	O too high	O too high	O too high

13) How well were you able to integrate the study into your day-to-day work?

O very well
O good
O rather poor
O poorly

Please briefly explain your assessment (free text)

____
____
____

14) How difficult was it for you to conduct the study in your institution in appropriate premises (with regard to the privacy of the participants)?

O very easy
O easy
O difficult, but feasible
O not possible at all

Please briefly explain your assessment (free text)

____
____
____

15) Can you imagine your organisation taking part in a regular data collection like this?

O definitely
O possibly
O not assessable
O rather not
O not at all

Please give brief reasons for your assessment and, if applicable, indicate any necessary requirements for the possibility of regular participation (free text

____
____
____

Recruitment process

16) Approximately how many patients/clients have injected drugs in your organisation in the last 12 months during the study?

_________________ clients

O I don’t know

17) Were certain groups of people more likely to be invited to participate in the study than others (e.g. people of certain origins due to the availability of interpreters, people with good German language skills due to the lack of interpreters, people at high risk of infection, etc.)?

O yes
O no

If yes, which one and why? (Free text)

____
____
____

18) How willing were the invited patients/clients to participate?

O very good
O good
O rather poor
O poorly

Please briefly explain your assessment of their willingness to participate (free text)

____
____
____

19) In your opinion, why did the invited patients/clients from your institution take part in the study? (free text)

____
____
____

20) How would you rate the study information and consent form with regard to the scope, comprehensibility and information content of the information provided?

**Scope**	**Comprehensibility**	**Information content for participants**
O too short	O very good	O very good
O adequate	O good	O good
O too long	O rather poor	O rather poor
	O poor	O poor

Comment if applicable (free text)

____
____
____

Questionnaire

21) How do you rate the questionnaire in terms of the scope and comprehensibility of the questions?

**Scope**	**Comprehensibility**
O good length	O easy to understand
O a bit too long, but feasible	O a bit complicated, but still understandable
O much too long	O too complicated, not understandable at all

Comment if applicable (free text)

____
____
____

22) Did the study participants themselves decide whether or not to complete the questionnaire?

O yes
O no

If no, why not? (free text)

____
____
____

23) Overall, how do you rate the feasibility for the study participants to complete the questionnaire in writing themselves?

O very good
O good
O rather poor
O poorly

Please briefly explain your assessment (free text)

____
____
____

24) If you assisted the participants in completing the questionnaire, what support did you have to give them?

O For most study participants, it was sufficient to have the questions and answer options read out to them
O Most study participants needed additional explanations of the questions and answer options
O Most study participants did not understand many questions despite the explanation

25) How do you rate the time required for assisted completion of the questionnaire?

O not at all high
O not very high
O a little too high
O much too high

Laboratory

26) How do you rate the (venous) blood collection (and the dripping of the filter cards)?

**Time required**	**Feasibility**	**Acceptance by participants**
O not at all high	O very good	O very good
O not very high	O good	O good
O slightly too high	O rather poor	O rather poor
O much too high	O poor	O poor

Comment if applicable (free text)

____
____
____

Only for study arm 2

27) How do you rate the implementation of the rapid tests?

O very feasible
O feasible
O not very feasible
O not feasible at all

Please briefly explain your assessment (free text)

____
____
____

Only for study arm 2

28) In your opinion, how well did the participants accept the rapid test offer?

O very good
O good
O rather poor
O poor

Only for study arm 2

29) If participants have not accepted the rapid test offer, what do you think are the reasons for this? (free text)

____
____
____

Only for study arm 2

30) In your opinion, did the participants accept the rapid test offer as a substitute for returning the test results?

O yes, there was no or hardly any criticism
O fair, there was criticism from some
O no, there was a lot of criticism

Only for study arm 3

31) How difficult was it in your institution to organise the return of the results from the laboratory test?

O very easy
O easy
O difficult, but feasible
O not possible at all

What were the difficulties, if any, in organising the return of the test results? (free text)

____
____
____

Only for study arm 3

32) Approximately how many participants collected their test results?

O 75-100%
O 50-74%
O 25-49%
O 0-24%
O We are unable to estimate

Only for study arm 3

33) If participants have not collected the results, what do you think are the reasons for this? (free text)

____
____
____

Only for study arm 2&3

34) Approximately how many participants with a positive (rapid) test result could be referred to specialized medical treatment?

O 75-100%
O 50-74%
O 25-49%
O 0-24%
O We are unable to estimate

What were the reasons why you were unable to offer a referral? (free text)

____
____
____

Only asked in Bavaria

35) How high do you estimate the proportion of participants from your organisation without health insurance valid for Germany?

O 75-100%
O 50-74%
O 25-49%
O 0-24%
O We are unable to estimate

Translations and language mediation

36) Were there any languages missing for which you would have needed a translation of the study document?

O yes
O no

If yes, which languages were missing? (free text)

____
____

37) Did you use language mediation?

O yes
O no

If no, why not? (free text)

____
____
____

If you used language mediation: (otherwise please continue to question 40)

38) Did you experience any problems?
O no, no problems
O yes, the following
O no one reached
O required language not available
O study process took too long with language mediation
O Filling out the documents (especially the questionnaire) was difficult with language mediation
O Conversation with language mediation was generally difficult
O Other problems: ____
____

39) How helpful was the language mediation for you?

O very helpful
O conditionally helpful
O not helpful

Please briefly explain your assessment of the language mediation? (free text)

____
____
____

40) If you did not use language mediation, please briefly explain why not (free text)

____

____

____

41) Were you able to recruit study participants through the translated study documents and/or language mediation that you would not have reached otherwise?

O yes
O no
O I don't know

Incentive

42) How would you rate the incentive given to the study participants?

**Amount**	**Form**	**Acceptance by participants**
O too high	O voucher is good	O overall well accepted
O appropriate	O voucher is accepted okay	O overall acceptance was okay
O too low	O voucher is poor	O overall acceptance was poor
	O we gave out cash	

Comment if applicable (free text)

____

____

____

Other comments

43) Do you have any comments on the study programme?

____

____

____

Thank you very much for your help. The questionnaire has now been completed.
