# Supplementary material for: Acceptance and feasibility of a low-threshold and substitution services-based periodical monitoring system for blood-borne and sexually transmitted infections among people who inject drugs in Germany: a mixed-methods analysis
Source: Harm Reduct J. 2024 Mar 14;21:62. doi: 10.1186/s12954-024-00977-0 (PMC10938743; doi:10.1186/s12954-024-00977-0)
Supplement: Supplementary file 2 — Additional file 2: Interview guide. [file 12954_2024_977_MOESM2_ESM.pdf]

DRUCK 2.0
Drogen und chronische Infektionskrankheiten – Pilotstudie

ROBERT KOCH INSTITUT

Interview guide group discussions – Facilities (translated from German)

Explanation of the procedure, consent to audio recording

Introduction of the organisation: How many employees does your organisation have? How many were involved in the study?

1) **Why did your organisation take part in the DRUCK study?**
2) **What are your experiences?**

   What worked well?

   Where were the hurdles?

   The following points and aspects can also be asked specifically during the interview:

   a. Recruitment & information
      i. Is the location/facility suitable for recruitment?
      ii. Is the location suitable for implementation?
   b. Questionnaire
      i. Length?
      ii. Too private?
      iii. Completed alone/assisted?
   c. Language mediation (Language mediation was welcomed at the beginning, but has not been used so far. As this service is very expensive, it would be important for us to know why not).
      i. Need?
      ii. Hurdles to utilisation?
      iii. Attempt but problems with accessibility?
   d. Testing
      i. Inhibitions during blood sampling?
      ii. Too many spots on the cards that need to be dripped?
   e. Return of results, if applicable
      i. Could the results be returned?
      ii. How time-consuming was this? (Documentation contact list, presence of doctor)
      iii. In the case of rapid testing: How did the post-test counselling go?
   f. Transfer to treatment:
      i. Are there contacts for referral?
      ii. Were participants who tested positive successfully referred?

g. If applicable, from the participants' point of view?
    i. Were there any hurdles on the part of the participants that you are aware of?
    ii. Or aspects of the study that the participants found good or bad?

If it is not addressed anyway, the study programme for the months of October - April could also be addressed here:

a. Study preparation/training
    i. Did you feel well prepared?
    ii. Which areas need more preparation?
    iii. Were you able to remember the content you needed even after a long time?
    iv. Was the refresher meeting in January helpful for the rest of the recruitment period?
b. Study implementation and possible changes over Christmas
    i. Have the conditions for conducting the study in your organisation changed over the period of the study?
    ii. How useful do you think the winter months are for conducting the study?
c. Extension (April)
    i. To what extent was the extension of the recruitment period in April helpful for you?
d. Continuous testing vs. test week models
    i. How did you plan the implementation in your organisation?
    ii. To what extent did this model work?
    iii. Which model do you think makes more sense?
e. Cooperation partners (only applies to organisations with cooperation partners)
    i. Which tasks were taken on by which partners?
    ii. How helpful was this for the realisation of the study?
    iii. Would such co-operation also be conceivable in the future?
    iv. Would it be possible to carry out the study without this cooperation?

3) What would have to be different for data collection of this kind to be possible in your institution?

4) What hurdles should the RKI help you overcome? And how?

5) Based on your current experience, to what extent do you think monitoring ...

a. ... makes sense?
b. ... is feasible?

6) What would monitoring have to look like for it to work?

If no ideas come up, go through the following points or work on the previously mentioned aspects/hurdles and find possible improvements.

a. Recruitment & education
   i. Where and how can potential study participants be reached and recruited in the best possible way?

b. Questionnaire
   i. How long should the questionnaire be?
   ii. Comprehensibility?
   iii. What could be helpful when completing the questionnaire?
   iv. Do you think it makes sense for participants to complete the questionnaire on their own?

c. Testing
   i. Once again the fundamental question: Should results be returned or not?
   ii. Would a quick test be an adequate alternative for returning results?
   iii. How many drops are realistic? (13 drops were required in Bavaria, 17 drops in Berlin)

d. Return of results
   i. Follow up on the previous and general question on the return of results.
   ii. How could participants be better reached for the return of results?
   iii. What could be improved for the post-test counselling (as mainly institutions from study arm 2 will be there)?

e. Study preparation/training
   i. Would it also be conceivable to carry out the study preparation independently online (videos)?
   ii. What would the study preparation ideally have to look like for the institutions so that it is feasible in terms of time and personnel?

Interview guide group discussions – Study participants (translated from German)

Explanation of procedure

Note that no personalised aspects are expressed, i.e. it is not about your own information or test results

Consent to audio recording

1) **Why did you take part in the study?**
2) **What did you like and dislike about taking part in the study?**

   If necessary, go through individual aspects of the study design

   a. Importance/content of the study (clear what the study was about?)
   b. Survey & questionnaire
      i. Did you complete the questionnaire yourself?
      ii. Was it important for you to complete the questionnaire with someone else?
   c. Blood collection
      i. How did you feel about the blood collection?
      ii. Was the amount of blood okay?
      iii. (Could you imagine dripping/drawing the blood yourself?)
   d. Rapid test, if necessary
      i. Have you carried out the rapid tests?
      ii. Did you find the rapid tests helpful?
   e. Collection of results, if applicable
      i. Did you collect your result?
      ii. Did you find the rapid tests helpful?
   f. (Incentive)
      i. Was the incentive in the form of a voucher okay?
3) **Would you participate in this study again?**
   a. What could be better or what should be different?
   b. Would you participate again if there were no results or rapid tests?
4) **Is there anything else to add?**
